# Development and Optimisation of Solid-Phase Extraction of Extractable and Bound Phenolic Acids in Spelt (*Triticum spelta* L.) Seeds

**DOI:** 10.3390/antiox10071085

**Published:** 2021-07-05

**Authors:** Marjeta Mencin, Maja Mikulic-Petkovsek, Robert Veberič, Petra Terpinc

**Affiliations:** 1 Department of Food Science and Technology, Biotechnical Faculty, University of Ljubljana, Jamnikarjeva 101, SI-1111 Ljubljana, Slovenia; marjeta.mencin@bf.uni-lj.si; 2 Department of Agronomy, Biotechnical Faculty, University of Ljubljana, Jamnikarjeva 101, SI-1111 Ljubljana, Slovenia; maja.mikulic-petkovsek@bf.uni-lj.si (M.M.-P.); robert.veberic@bf.uni-lj.si (R.V.)

**Keywords:** spelt phenolics, extraction, hydrolysis, solid-phase extraction, liquid-liquid extraction, antioxidant activity, LC-MS

## Abstract

A solid-phase extraction (SPE) technique was developed and optimised for isolation and concentration of extractable and bound phenolic acids from germinated spelt seeds, for analysis by liquid chromatography–mass spectrometry. Samples initially underwent solvent extraction under different conditions to maximise the yield of phenolic antioxidants. Optimal extraction conditions for extractable phenolics were absolute methanol as solvent, sample-to-methanol ratio 1:9, and reconstitution in non-acidified water. The bound phenolics were extracted from sample pellets using hydrolysis with 2 M NaOH, acidification of the hydrolysate with formic acid, and simultaneous isolation and purification using Strata X polymeric RP tubes. Compared to liquid-liquid extraction, this direct SPE protocol has significant advantages in terms of higher extraction efficiencies of total and individual phenolics and their antioxidant activities. These data suggest that direct SPE represents a rapid and reliable method for quantitative analysis of both the extractable and the commonly overlooked bound phenolics in *Triticum spelta* seeds.

## 1. Introduction

Spelt (*Triticum spelta* L.) is an ancient form of wheat, and it is cultivated in several central European countries. Over the past few decades, it has attracted renewed interest as a healthier, more natural, less ‘over-bred’ cereal compared to modern common wheat (*Triticum aestivum* L.). More recently, this has been combined with increased attention on the phenolics in the whole grain of *Triticum* species [1,2,3]. Our previous study suggested that germination of spelt seeds under specific combined stress conditions can significantly improve their total phenolics content (TPC), along with the levels of the individual phenolics and their scavenging activities against different free radicals (e.g., DPPH^•^, ABTS^•+^, O_2_^•−^, ROO^•^) [4].

Phenolics can occur as soluble free phenolics in the vacuole of plant cells, while their soluble conjugates are covalently bound or esterified to sugars and other low molecular mass components. These soluble phenolics are often referred to as ‘extractable’, and they are generally extracted from food matrices using different combinations of aqueous and organic solvents. However, phenolics can also be covalently bound to cell-wall materials through ester, ether and carbon–carbon bonds, or entrapped in the macromolecules of food matrices through hydrophobic interactions and hydrogen bonding [5]. These represent the insoluble ‘bound’ phenolics.

Similar to other cereals, these bound phenolics of the grain of modern and ancient varieties of wheat mostly remain in the solid residues after conventional solvent extraction. Their quantification can be improved using acidic [6], alkaline [7] and enzymatic [8] hydrolysis. Zhang et al. [5] recently highlighted the roles for *Triticum* phenolics in human health, and therefore a suitable extraction method for obtaining these bound components is much in demand, especially as they have often been ignored.

A wide range and combination of techniques have been used to isolate and purify these extractable and bound phenolics from the wheat matrix [9]. For example, solid-phase extraction (SPE) is a popular method that uses a solid phase and a liquid phase to isolate analytes from solutions. SPE has usually been used as a ‘clean-up’ procedure prior to chromatographic or other analytical methods that can then be used to quantify various analytes. SPE based on reversed-phase polymeric sorbents allows the extraction of phenolics and the removal of sugars and other highly polar compounds (e.g., organic acids, amino acids, proteins) [10]. Many studies have focused on the extraction and SPE purification of the extractable phenolics from cereals [11,12,13,14,15] and reported that SPE clean-up step allows high recoveries and good precision for extractable phenolic acids in a different cereals. To the best of our knowledge, the application and optimisation of SPE for the extraction and purification of bound phenolics extracts has not yet been reported. The optimisation of extraction and SPE purification for particularly bound phenolics would be helpful for their more routine analysis.

liquid-liquid extraction (LLE) can also be used to enrich high-molecular-weight phenolics and to clean-up extracts using suitable solvents [16]. LLE is one of the most widely used technique applied to extraction of phenolics from different cereals [17,18,19,20]. Although LLE is inexpensive, it involves the use of organic solvents (e.g., often highly toxic diethyl ether, either alone or in combination with ethyl acetate) and requires longer extraction times. This can also result in extract degradation.

Although the equipment required for SPE is more expensive than for LLE, the use of SPE can avoid many of the problems associated with LLE, such as incomplete phase separations, unsatisfying recoveries, use and disposal of large quantities of organic solvents, among others [21].

The main objective of the present study was to develop an extraction and purification protocol for analysis of spelt seed phenolics, to facilitate their isolation for subsequent characterisation and quantification, and to also overcome the drawbacks of the previously established LLE method. We strongly believe that the direct SPE method presented here will also be useful for other *Triticum* species, as they are genetically and anatomically very similar to spelt.

## 2. Materials and Methods

### 2.1. Materials

Spelt (*Triticum spelta* L. cv. Ostro) seeds were obtained from a local mill in the Dolenjska region of Slovenia. The seeds were stored in the dark at 1 °C until analysis. The preparation of the germinated spelt seeds was reported in detail by Mencin et al. [4].

Methanol (99.9%), formic acid, sodium hydroxide and sodium carbonate were from Merck (Darmstadt, Germany). 2,2-Azino-bis-3-ethylbenzothiazoline-6-sulfonic acid (ABTS) reagent, the 2,2-diphenyl-1-picrylhydrazyl radical (DPPH^•^) reagent, Folin–Ciocalteu reagent, Trolox, sodium dihydrogen phosphate dehydrate, *p*-hydroxybenzoic acid, *trans*-ferulic acid, *p*-coumaric acid and caffeic acid were from Sigma-Aldrich (Steinheim, Germany). All of the chemicals and reagents used for the present study were of analytical quality. Water (Milli-Q; Millipore, Billerica, MA, USA) was used to prepare the working solutions.

### 2.2. Preparation of Crude Extractable and Bound Fractions

In recent years, a lot of research has been carried out on germinated grain, with aim to improve the nutritional value. Therefore, we optimised the following methods for use with germinated seeds.

For the preparation of crude extractable and bound fractions, 1.0 g germinated seeds were milled (A11 basic; IKA-Werke GmbH, Staufen im Breisgau, Germany) and freeze-dried (VirTis AdVantage Pro Freeze Dryer, SP Scientific, Gardiner, NY, USA) in different volumes of water or methanol, and left shaking (EV-403; Tehnica, Železniki, Slovenia) for 2 h. The initial investigations indicated that pure methanol provided greater extraction for the more prevalent phenolic acids in the spelt seeds, and therefore the further optimisation was carried out with only methanol as the extraction solvent.

The aliquots of the spelt seeds were mixed with 99.9% methanol at a ratio of freeze-dried seeds to methanol of 1:9 or 1:6 (*w/v*). After 2 h of extraction at room temperature, the mixtures were centrifuged at 9793.9× *g* for 10 min. The supernatant was then removed as the ‘crude extractable fraction’, and the residue was processed further for the bound fraction.

### 2.3. Preparations for Direct Solid-Phase Extraction of Phenolics

#### 2.3.1. Preparation of Extractable Phenolics for Solid-Phase Extraction

**Figure 1:**
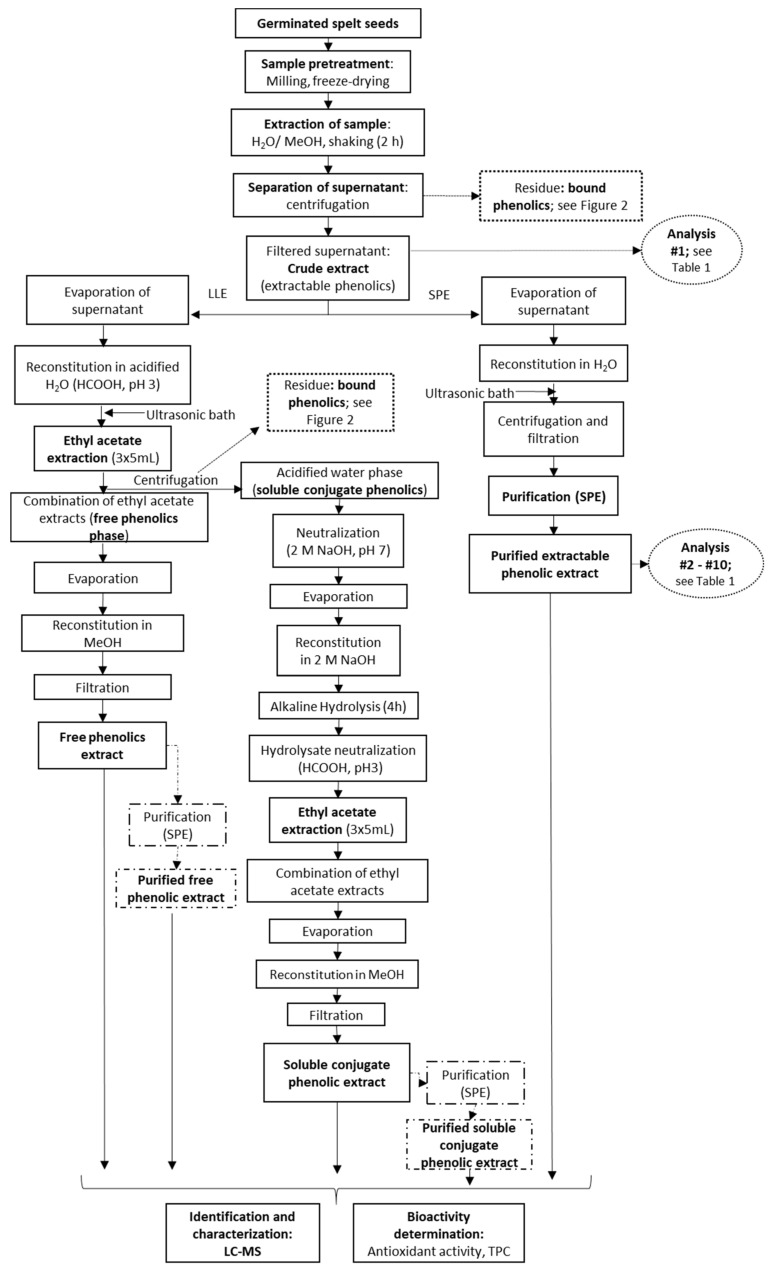
Processing of the germinated spelt (*Triticum spelta*) seeds to obtain the extractable phenolics through direct purification by solid-phase extraction (SPE) and the free and soluble conjugate phenolics through liquid-liquid extraction (LLE) without and with SPE purification. For analyses indicated, see Table 1.

Following filtration of the crude extractable phenolics fraction (pore size, 0.45 µm, Macherey-Nagel, Duren, Germany), this was further processed for direct SPE according to one of two different procedures. For the first, an aliquot of the supernatant was diluted to 10% methanol with water, and then applied to an SPE column. For the second, the solvent was evaporated off an aliquot of the supernatant (5 mL) in a vacuum evaporator (HT-4 series II; GeneVac Technologies, Ipswich, UK), and the residue was re-suspended in 5 mL water (pH 7, if not specified otherwise) (Figure 1). The samples were then filtered and applied to an SPE column.

#### 2.3.2. Preparation of Bound Phenolics for Solid-Phase Extraction

**Figure 2:**
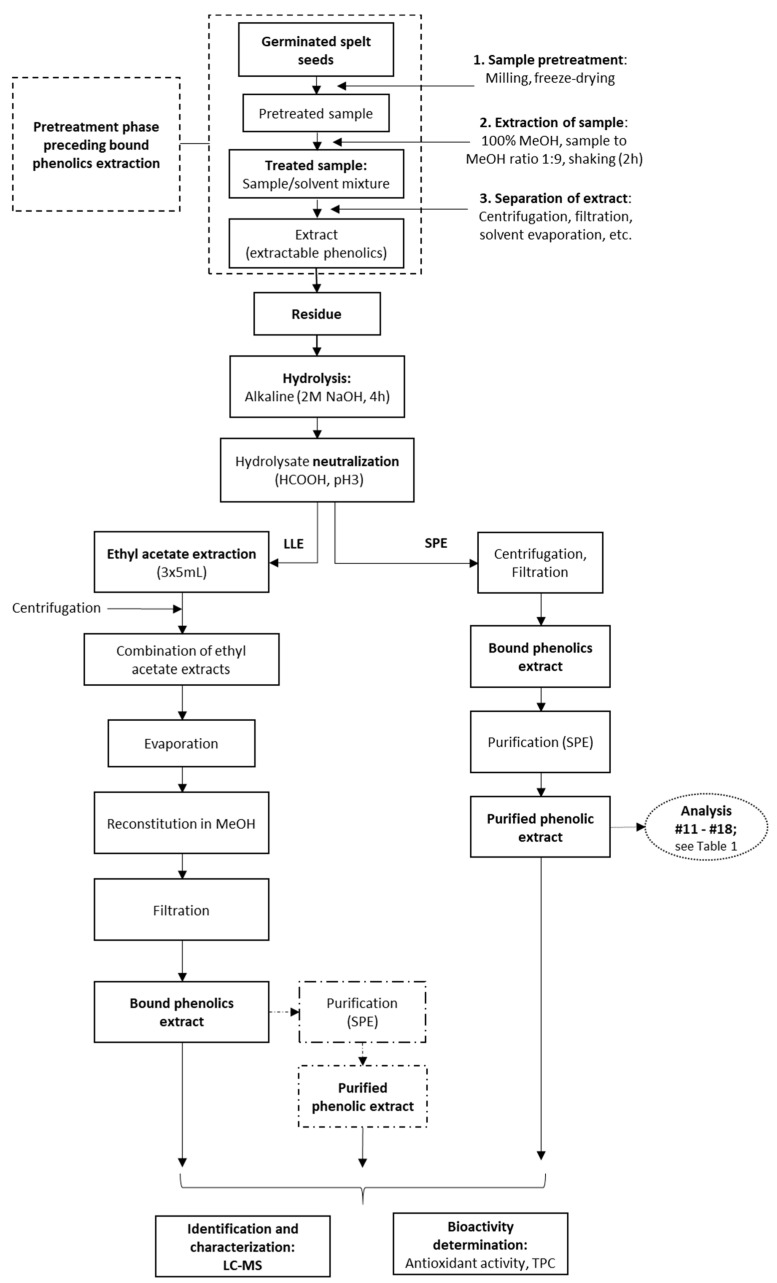
Processing of the germinated spelt (*Triticum spelta*) seeds to obtain the bound phenolics through direct purification by solid-phase extraction (SPE) and through liquid-liquid extraction (LLE) without and with SPE purification. For analyses indicated, see Table 1.

After the removal of the supernatant from the methanol extraction of the spelt seeds (i.e., the ‘crude extractable phenolics’), the solid residue was treated with 2 M sodium hydroxide (NaOH; 10, 15, 20 mL) with shaking for 4 h (EV-403; Tehnica, Železniki, Slovenia) at room temperature. After this alkaline hydrolysis step, the hydrolysed sample that contained the previously bound, but now released, phenolics (i.e., the ‘crude bound phenolics’) was acidified to pH 2 with 6 M HCl, or to pH 3 with concentrated formic acid (Figure 2). These samples were then filtered (pore size, 0.45 µm; Macherey-Nagel, Duren, Germany) and loaded onto an SPE column.

### 2.4. liquid-liquid Extraction of Phenolics

#### Preparation of Free Phenolic Extract

The solvent from 5 mL of the supernatant for the crude extractable phenolics was evaporated off, and the dry methanolic extract was redissolved in 5 mL water acidified with formic acid (pH 3). This was then extracted three times with ethyl acetate (5 mL) for 5 min. The combined ethyl acetate fractions were evaporated to dryness at 30 °C, and the residue was reconstituted in methanol and filtered through a 0.45 μm membrane filter (Chromafil A-45/25 syringe filters; cellulose acetate, hydrophilic membrane; Macherey-Nagel, Duren, Germany). This provided the LLE ‘free phenolics fraction’ (Figure 1). For additional SPE purification of the ethyl acetate extracts (i.e., LLE + SPE), the residue following evaporation was reconstituted in water instead (Figure 1).

### 2.5. Solid-Phase Extraction

The SPE tubes Strata-X polymeric RP 3 mL/100 mg (Phenomenex, Torrance, CA, USA) were initially preconditioned with 3 mL 99.9% methanol, followed by equilibration with 3 mL water, or water acidified with HCl or formic acid (dependent on solvent used to provide the dried methanolic extracts). The samples that contained the extractable, free, soluble conjugate or bound phenolics were then added onto the SPE columns with the liquid allowed to enter the tube matrix. The tubes were then washed with 4 mL water and vacuum-dried for 2 min. Finally, the target compounds were eluted with 2 mL 70% aqueous methanol. The eluates obtained represented the corresponding purified extractable, free, soluble conjugate and bound phenolics.

### 2.6. Determination of Total Phenolics Content

The TPC was determined using the Folin–Ciocalteu method, according to the protocol described by Mencin et al. [4]. A standard curve was prepared with Trolox, and the data are expressed as mg Trolox equivalents per g dry weight (mg TE/g DW).

### 2.7. Liquid Chromatography–Mass Spectrometry Analysis

All the instruments and analysis conditions for the liquid chromatography–mass spectrometry (LC-MS) were the same as previously reported by Mencin et al. [4]. Identification of the individual phenolics was confirmed by comparisons of retention times and spectra with standard databases, and by addition of standards to samples, followed by fragmentation of each component (Supplementary Table S1). The contents of *p*-coumaric acid, *trans*-ferulic acid, caffeic acid derivatives and *p*-hydroxybenzoic acid were calculated from the peak areas of the samples and the corresponding standards and are expressed as µg per g dry weight (µg/g DW) of the germinated spelt seeds.

### 2.8. HPLC Method Validation

Calibration, limits of detection (LOD), limits of quantification (LOQ), linearity and accuracy were measured for method validation. Linearity was evaluated by analysing mixtures of phenolic acid standard solutions and by spiking samples with known amounts of various analytes. A calibration curve was constructed separately for each compound by plotting peak area vs. concentration. The calibration curve was fitted by linear least-squares regression, and the value obtained for the correlation coefficient showed that the method is linear in the concentration range studied.

Accuracy was evaluated through the percent recovery of the phenolic compounds calculated by spiking the sample with known amounts of the compounds at three different concentrations (low, medium, high).

The calculations for the LOD were calculated using the following equation LOD = 3.3 σ/S and the LOQ were calculated using the equation LOQ = 10 σ/S; where σ is the standard deviation of the response and S is the slope of the calibration curve. The calibration equations, correlation coefficient, LOD, LOQ and the average of recovery of each phenolic acid in spiked samples are compiled in Supplementary Materials Table S1.

### 2.9. DPPH^•^ Scavenging Activity

The antioxidant activities of the phenolics from the germinated spelt seed extracts were determined using DPPH^•^ [22], according to Mencin et al. [4]. The antioxidant activities using DPPH^•^ (referred to as DPPH in Figures and Tables) are expressed as mg TE/g DW.

### 2.10. ABTS^•+^ Scavenging Activity

The scavenging activities of the phenolics from the spelt extracts were determined using ABTS^•+^, according to the method described by Mencin et al. [4]. The antioxidant activities using ABTS^•+^ (referred to as ABTS in Figures and Tables) are expressed as mg TE/g DW.

### 2.11. Statistical Analysis

All of the analyses were performed for the data obtained in three parallel runs, on two separate extractions. The data are means ± SD. One-way analysis of variance (ANOVA) was performed using the SPSS programme (Version 22 for Windows). The comparisons of the treatment means were based on Duncan‘s multiple range tests, for a significance level of 0.05.

## 3. Results and Discussion

### 3.1. Effects of Extraction Parameters on Extractable Phenolics

The extraction yields of the phenolics from such plant tissues are affected by several parameters, including extraction technique, time, temperature and solvent-to-sample ratio, and number of repeated extractions and type of solvent [23]. Although the phenolic profiles of the crude samples showed high levels of the phenolic acids analysed here (Table 1), these samples need to be purified before LC-MS analysis. Purification using SPE can prevent impurities from accumulating on the analytical HPLC column. When a column becomes contaminated or blocked up with impurities, the pressure increases and the peaks broaden or split. The food matrix is generally a problem in any analytical technique, so there is the need to remove the contaminants so that they do not block up the column. Additionally, the crude samples showed broader peaks (Figure S1) compared to the purified samples, which indicated that better separation between the peaks can be achieved with the purification step. In the crude sample, the components were present at a much higher concentration, so the peaks begin to overlap the neighbour component at a lower concentration.

According to Stalikas [24], very polar phenolic acids cannot be extracted completely with pure organic solvents and mixtures of alcohol-water are instead suggested. Therefore, the extraction efficiency for the extractable phenolics was further examined with different proportions of methanol:water. Here (data not shown), the recovery of all of the analytes was the highest when pure methanol was used, where compared to the extraction with water, extraction with methanol resulted in 4% increase in TPC and 16% increase in the DPPH^•^ scavenging activity of the purified extractable fraction (Table 1). The type of solvent also had an impact on the phenolic profiles of these direct SPE-purified extracts. As can be seen from Table 1 (dataset #2, #3, #4), *p*-hydroxybenzoic acid was the predominant phenolic acid detected in the extractable fraction regardless the extraction solvent. Compared to extraction with the more polar water, extraction with methanol resulted in higher contents of *p*-coumaric acid (10%), *trans*-ferulic acid (14%) and *p*-hydroxybenzoic acid (32%) (Table 1, water 1:9 vs. methanol 1:9; #3 vs. #2). Interestingly, the content of the caffeic acid derivatives (22%) was significantly higher in aqueous extracts than in methanolic extracts (Table 1).

Among these phenolic acids studied here, caffeic acid is the most polar, because its two hydroxyl groups increase its hydrophilicity, which will be why it was better extracted with the more polar solvent, water. *p*-Coumaric acid is more hydrophobic than caffeic acid, followed by ferulic and *p*-hydroxybenzoic acid. This is reflected in the data here, where the impact of solvent polarity on extraction efficiency of individual phenolic acids increased with decreased polarity of the target molecules. According to Terpinc et al. [25], caffeic acid also has significantly greater Folin–Ciocalteu reducing capacity and DPPH^•^ radical scavenging ability than ferulic and *p*-coumaric acids. All of these aspects can partly explain the deviations among the TPC and the antioxidant activities of the individual phenolic acids determined in the present study.

**Figure 3:**
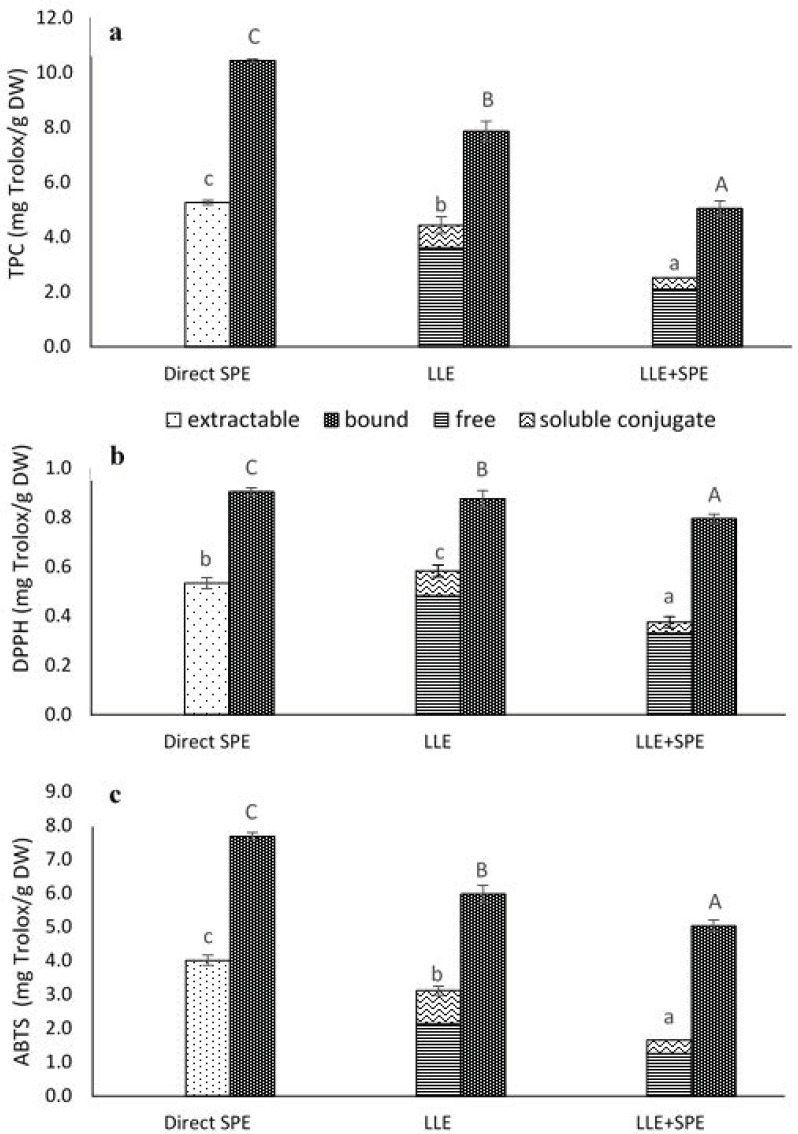
Total phenolics content (TPC) (**a**) and antioxidant activities (DPPH^•^, ABTS^•+^ scavenging activities) (**b**,**c**) from the extractions of the germinated spelt (*Triticum spelta*) seeds, as the extractable and bound phenolics from direct solid-phase extraction (SPE), and for the free, soluble conjugate and bound phenolics from the liquid-liquid extraction without (LLE) and with SPE (LLE + SPE) purification. Data are means ± SD. Different letters within the same fractions as extractable and (free + soluble conjugate) or bound indicate significant differences (*p* < 0.05; Duncan’s multiple range tests).

Soluble conjugates are compounds with multiple hydroxyl groups, which increase the hydrophilicity of attached phenolic compounds, especially flavonoids [26]. According to the data shown in Figure 3, the methanolic extracts contained low levels of soluble conjugates. The prevalent free phenolics (within the extractable fraction) have a higher affinity for methanol compared to water. As can be seen from Table 1, TPC was less affected by the extraction solvent than the content of the individual phenolics, which suggests that the aqueous extracts had different compositions, where the phenolic acids were not the main reductants of the phosphomolybdate in the Folin–Ciocalteu reagent. It should be noted that both crude extracts were dried to dryness, reconstituted in dH_2_O and applied to SPE tubes as described in Section 2.2.

The recoveries of *p*-coumaric acid, *trans*-ferulic acid, caffeic acid derivatives and *p*-hydroxybenzoic acid after the purification step when methanolic extract was applied on the SPE tube were 93.49%, 94.00%, 95.54% and 89.51%, respectively (Supplementary Table S1). These data all indicate better selectivity of methanol than water for the extraction of the phenolic acids from spelt.

In addition, the extraction techniques need to take into account the location of the phenolic acids in the plant tissues. However, methanol can disrupt cell walls and inhibit enzyme activities, and it is a good solvent for most phenolics. The exceptions here are the phenolics that are bound to insoluble carbohydrates and proteins within the plant matrix [23]. However, various reports in the literature have shown that the total phenolics that can be extracted with polar solvents (e.g., water, methanol, ethanol) can vary considerably, depending on the sample matrix used [27].

As the yields of the total and individual phenolics were higher in methanol, the further optimisation was carried out with methanol only. The sample-to-methanol ratios of 1:6 and 1:9 were also tested with methanol extraction. When compared to the sample-to-solvent ratio of 1:6, that of 1:9 resulted in a 36% increase in TPC (Table 1, #2 vs. #4) and 14% increase in DPPH^•^ scavenging activity (Table 1). These data demonstrate that the higher proportion of methanol (i.e., 1:9) was better for increased reduction capacity according to Folin–Ciocalteu reagent and greater DPPH free radical scavenging activity. Increasing the proportion of methanol in the extraction will provide a greater concentration gradient during its diffusion, and thus the analytes will have a greater tendency to leave the matrix and move into the liquid phase [28]. However, too high a sample-to-solvent ratio coincides with higher solvent consumption following the extraction and energy consumption for concentration in a later processing stage [28]. It should be stressed here that the optimisation of these particular parameters was performed to maximise the adsorption of the target molecules (i.e., the phenolics) onto the SPE tube matrix.

As can be seen in Table 1, the higher sample-to-solvent ratio of 1:9 had a negligible impact on the extraction yield of most of the individual phenolic acids, expressed per unit mass of the sample matrix. However, *p*-hydroxybenzoic acid did show a statistically significant positive impact (11%), and so 9 mL of solvent was used per gramme of seeds for the further analyses, with larger sample volumes also needed for further analysis.

### 3.2. HPLC Method Validation

The calibration data for each standard phenolic acid as well as the LOD and LOQ for the spelt sample spiked with phenolic acid standards are shown in Table S1. As can be seen, the linearity of all compounds is satisfactory with R^2^ values > 0.9911.

The proposed method was found to be suitable and reliable for the determination of phenolic compounds as the recoveries ranged from 89.51% to 95.54%.

The LOD determined in spelt seed samples ranged from 0.094 to 0.524 µg/g, with the lowest and the highest values for *trans*-ferulic acid and caffeic acid, respectively. Furthermore, the LOQ values of the studied phenolic acids varied from 0.285 µg/g for *trans*-ferulic acid to 1.586 µg/g for caffeic acid (Supplementary Table S1).

### 3.3. Effects of the Extraction Parameters on Bound Phenolics

Wholegrain *Triticum* seeds are an excellent source of bound phenolic acids (which represent >90% of the total phenolic acids), followed by bound flavonoids and other phenolics [2,4,7,29,30,31]. As reported by Balli et al. [32], the hydrolysis conditions can significantly affect the total amounts and profile of the bound phenolics than can be extracted. The type of base/acid, the solid-to-solvent ratio, and the extraction method determine the amounts and type of phenolics released, and the antioxidant activities of the extracts obtained. In general, an increased solvent volume for the hydrolysis increases the extraction efficiency, while decreasing the solvent volume lowers the extraction efficiency due to saturation effects, although this will also decrease the cost. Depending on the method used to release and assess the bound phenolics, the conditions applied might be destructive or inefficient, thus causing degradation or incomplete release of the bound phenolics [5].

Most of these studies have used alkaline hydrolysis with NaOH for the release of the bound phenolics from *Triticum* seeds, while other studies have obtained higher amounts of phenolics using acid hydrolysis with H_2_SO_4_ or HCl [9]. In general, esterified phenolic acids (i.e., those linked to the cell wall polysaccharides by ester bonds) are more efficiently liberated by alkaline hydrolysis, while acid hydrolysis is more recommended for the release of phenolic acids from glycosylated forms (i.e., those linked to the solubilising sugars by ether bonds) [23]. While acidic pH and high temperature might result in degradation of some phenolics, on the other hand, after acidic hydrolysis, the extracted bound phenolics can be directly injected into a chromatographic system for further analysis after neutralisation and filtration. In contrast, alkaline hydrolysis requires an additional extraction procedure using diethyl ether or ethyl acetate [5].

In continuing this study, we next determined how the solid (residue)-to-NaOH ratio affected the efficiency of the later phenol binding to the SPE columns. The range of solid-to-liquid ratio selected for the alkaline hydrolysis in the present study was based on frequently documented literature data. Here, greater accessibility of the analyte to NaOH should result in increased levels of phenolics released. At the same time, we investigated what a highly concentrated (*versus* more dilute) hydrolysate obtained under the same experimental conditions would mean for the binding and elution efficiency of the phenolics obtained once applied to the SPE purification. Therefore, the seed residues from the methanol extraction had different volumes of 2 M NaOH added (10 mL, 15 mL, 20 mL; Table 1, dataset #11, #12, #13), to determine the effects of the added alkali on the levels of bound total and individual phenolics, and on the antioxidant activities of the extracts obtained.

As can be seen in Table 1, the SPE purified samples with higher bound TPCs also expressed higher antioxidant activities. Comparing the volumes of alkali added, as 10 mL vs. 20 mL NaOH, the larger volume resulted in 23% higher TPC and 16% greater DPPH^•^ scavenging activity. However, there were no significant differences between 15 mL and 20 mL NaOH for TPC, although 15 mL NaOH provided 9% greater DPPH^•^ scavenging activity compared to 20 mL NaOH, which reached statistical significance. In agreement with previous reports [6,24], different phenolic acids were extracted in different proportions depending on these hydrolysis conditions. Indeed, here the different seed residue-to-NaOH ratio (*w/v*; under the same purification conditions) had a large influence on the contents of the phenolic acids per unit mass of sample matrix. As can be seen in Table 1, comparison of the addition of 20 mL NaOH with that of 10 mL and 15 mL NaOH (respectively) resulted in the higher levels of *p*-coumaric acid (40%, 19%), *trans*-ferulic acid (70%, 35%), caffeic acid derivatives (209%, 67%) and *p*-hydroxybenzoic acid (61%, 21%). All of these phenolic acids increased linearly with increased volume of NaOH (R^2^ ≥ 0.98), which suggested a significant impact of solid-to-liquid ratio on the alkaline lability of these phenolic acids.

Thus, an increase in the sample-to-solvent ratio will lead to a greater concentration gradient during the extraction, which will promote the movement of the analytes from the matrix into the liquid phase [28]. The addition of 20 mL NaOH was therefore the optimal choice for all of the phenolic acids after SPE purification (expressed in DW), and was therefore incorporated into the protocol.

### 3.4. Optimisation of Solid-Phase Extraction Conditions for Extractable Phenolics

One of the aims of this study was to develop and optimise a direct SPE method for rapid and selective separation of the extractable phenolics prior to analysis by LC-MS. In the first step, the influence of sample preparation on the interactions between analyte and sorbent was investigated. When an analyte is extracted from a matrix by a sorbent, the selective removal of impurities can often be achieved by changing the polarity of the solvent [10]. Based on our preliminary data (data not shown), it was necessary to reduce the amount of methanol in the extracts obtained to optimised the binding capacity of the phenolics applied directly to the SPE tubes that contained Strata X-RP. Our first approach was dilution of the methanolic extract with water to avoid potential losses from evaporation and reconstitution. As shown in Table 1 (#5), application of 30 mL extract at 10% methanol resulted in lower yields of the total and individual phenolic acids compared to the more standard reconstituted sample (Table 1, #5 vs. #6). This suggested that when the 30 mL sample was added directly to the SPE tube, part of the phenolic acids flowed through the column without binding to the matrix, potentially due to a combination of excessive solvent application and too high a proportion of methanol.

Consequently, the second approach was that after preparation of the extractable phenolics, instead of dilution of the methanol, it was evaporated off and the dry residues were re-dissolved (i.e., reconstituted) in 5 mL water. In this step, if required, pH adjustment based on the analyte pKa is also recommended, to improve the liquid extraction efficiency. With the pKa values of the target phenolic acids here from 4.54 to 4.65, reconstitution was carried out with three different solvents: water, at pH 7; and acidic water, at pH 3 with formic acid or at pH 2 with HCl (Table 1, dataset #6, #7, #8). The same solvent in which the sample was dissolved was used to equilibrate the SPE column. The pH of the water used for the reconstruction had negligible effects on TPC and on the antioxidant activities (Table 1). Acidification of the water to pH 2 with HCl compared to water at pH 7 improved the TPC (9%) and compared to water at pH 7 and acidic water at pH 3, also increased the antioxidant activity (6%, 4%, respectively). There were no significant differences between TPCs of acidic water pH 2 and pH 3. According to these data, the pH of the reconstitution water affected only the extractable *p*-hydroxybenzoic acid content (Table 1). Lower yields in the SPE eluate were observed (expressed per unit mass of sample matrix) when the water reconstitution at pH 7 was applied directly to the SPE column, compared to the acidic water at pH 3 or pH 2 (27%, 29%, respectively). It would thus appear that acidification of the water and the subsequent protonation of the carboxylic groups is important only for efficient π-π interactions between the SPE tube matrix of the Strata X-RP sorbent and *p*-hydroxybenzoic acid (which has the shortest chain among these phenolic acids). Further, for the successful binding of other phenolic acids to the SPE sorbent, acidification of the water is not necessary.

Therefore, reconstruction in 5 mL water (pH 7) was selected as the standard reconstitution step here. This is not in agreement with Buszewski and Szultka [33], who reported that pH should be two units lower than the pKa of the analytes. A similar approach was adopted by Irakli et al. [11], who evaporated off their extraction solvent (70% methanol) from 2 mL of extract, followed by addition of 2 mL acidified water (1% (*v/v*) acetic acid, pH 2.6). The extraction efficiency of phenolic acids was further examined at different pHs of their loading solution (pH 2.6, 5.1). Here, they reported that the recovery of all of their analytes was increased by acidification of the loading solution to pH 2.6 when compared to pH 5.1, due to the suppression of the dissociation of the phenolic acids and to their more effective adsorption onto the tube sorbent.

Next for the reconstruction of these extractable phenolic acids, the effects of the loading volume on the extraction efficiency were studied. Here, with the extract reconstituted in 5 mL water (pH 7), two different volumes were then applied directly to the SPE tube: 3 mL and 5 mL (Table 1, dataset #9, #10). According to the data obtained, the higher SPE loading volume resulted in slightly, but significantly, higher TPC (Table 1, 4%) and DPPH^•^ scavenging activity (Table 1; 3%) of the SPE purified extracts. The extraction efficiency per unit mass of sample matrix also increased with the increase in the amount of sample loading for each of the extractable phenolic acids, *p*-coumaric acid (8%), *trans*-ferulic acid (13%), caffeic acid derivatives (9%) and *p*-hydroxybenzoic acid (11%). Therefore, a 5 mL loading volume was adopted as the isolation and purification procedure for these extractable phenolics.

To summarise to this point, this study has confirmed that the efficiency of the recoveries through the direct SPE purification is affected by the sample volume and the solvent composition (i.e., 10% methanol, water, acidic water) in which the sample is applied to the tube. According to these data, we believe that among these extractable antioxidants there is a large proportion of very polar antioxidants, which re-dissolve best in the neutral water, but which must first be fully liberated from the cells (with the methanol extraction).

Compared to the crude extract here, there were some losses in terms of the *p*-coumaric acid (37%), *trans*-ferulic acid (24%), caffeic acid derivatives (47%) and *p*-hydroxybenzoic acid (63%) from the direct SPE purification under this optimised sample processing: i.e., extraction with 99.9% methanol; sample-to-solvent ratio 1:9; reconstitution with water (pH 7); and 5 mL loading sample directly onto the SPE tube. We can conclude therefore that the sample preparation (i.e., evaporation of methanol, reconstitution in suitable solvent) before the direct SPE purification had a significant effect on the loss of these phenolic acids.

It can be stressed here that during the few hours of solvent evaporation, the most thermolabile compounds might undergo decomposition and lose their antioxidant activities. Moreover, some of the compounds successfully extracted from the germinated spelt might remain on the walls of the test tubes, and therefore incomplete dissolution might also contribute to poorer recovery. The various intermediate steps, such as centrifugation and filtration, will also affect the final recovery rates. These findings in the present study can be used as a basis for future studies, with a view to further optimisation of this protocol.

### 3.5. Optimisation of Solid-Phase Extraction Conditions for Bound Phenolics

Sample hydrolysis is a key step during the treatment of these samples, as this breaks down and releases the bound phenolics. In most studies, the extraction of the bound phenolics is completed with ethyl acetate or diethyl ether extraction after their hydrolysis (Figure 2). One of the main sources of errors during such analytical procedures is the sample preparation. It is crucial to shorten the procedures and to enhance the accuracy and selectivity for the identification of the majority of the chemical components here [34].

The principle of SPE is similar to that of LLE, in terms of the partitioning of the solutes between two phases (Figure 2) [10]. Generally, during the extraction process, an aqueous sample passes through an immobilised phase, and is afterwards extracted (or released) using a suitable organic solvent [33]. Therefore, instead of further solvent extraction here, the NaOH hydrolysate was applied to SPE to increase the yields of the bound phenolics in the extracts (Figure 2). The main objective of the SPE here was thus for removal of interfering matrix components, for concentration and isolation of the bound phenolics, and for changing the matrix of the analyte as needed for subsequent analysis.

In the present case, the phenolic acids must have a greater affinity for the sorbent that makes up the solid phase than for the sample (liquid) matrix. The relationships between the target compounds and the sorption during SPE include hydrophobic interactions (e.g., van der Waals forces) and hydrophilic interactions (e.g., dipole-dipole, induced dipole-dipole, hydrogen bonding, π−π interactions). Additionally, there are electrostatic attractions between the charged groups on the analyte of interest and the sorbent surface, along with molecular recognition mechanisms [33].

Separation of the analytes and the interfering compounds (i.e., impurities) using SPE can be realized by selective washing, starting with the compound of interest and the impurities being retained on the sorbent bed when the sample passes through. The impurities can then be rinsed off the sorbent bed with wash solutions that are strong enough to remove them, but weak enough to leave the compounds of interest behind [10]. As reported by Rodríguez et al. [35], the strategy to concentrate phenolics using a polystyrene–divinylbenzene sorbent include sorbent activation, concentration of the water sample (pH 2–3), drying of the sorbent bed, and elution using methanol or other solvents. As the liberated (alkaline hydrolysed) phenolics in the present study were already transferred into acidified water, no additional pretreatment appeared necessary. In Section 3.4. we also demonstrated that the reconstitution and equilibration of the extractable phenolic acids using water or acidified water showed negligible differences in the levels of total and individual phenolic acids following SPE.

Therefore, the reconstitution of the bound phenolics in non-acidified water would result in greater losses due to various intermediate steps (i.e., drying, reconstitution, filtration). As the liberated phenolics will have higher affinity towards the non-polar stationary phase due to their protonation, they will be adsorbed easily from an aqueous environment onto the non-polar polymeric sorbent. Finally, elution would be achieved by solvents with lower polarity. The SPE optimisation also involved the selection of a suitable washing and elution solution, which would provide the highest recovery rates, although this was not included in current study.

According to Rodríguez et al. [35], retention of phenolics is the result of a reversed-phase mechanism and π-π interactions among electrons from the aromatic rings in the sorbent and in the phenol molecules. This latter depends strongly on the properties of the crude extract. The correct choice of the reconstitution media (i.e., composition, pH) is therefore essential to obtain quantitative retention of phenolics.

Solvents with acidic pH are usually used for neutralisation of extracts after hydrolysis, as the phenolics are generally more stable at lower pH. Therefore, the degradation of the bound phenolics is lower at lower pH, compared to higher pH [30]. For extraction into organic solvents, low pH causes the phenolic group equilibrium to be shifted to fully protonated, and thus less polar, and more soluble in less polar solvents. This depends on other functional groups near the phenol group, in terms of whether they are electron withdrawing or donating, which can change the pKa of the phenolic group. In addition, other groups on the phenolic molecules might have their own pKa, which would change the solubility at different pHs. As already mentioned, if reversed-phase SPE is used for acidic analytes, the pH is usually adjusted to two units below the pKa of the target molecules.

In the present study, the influence of hydrolysate neutralisation with HCl or formic acid on TPC, antioxidant activity and the content of the individual phenolic acids were investigated. The results will define the preferred pH of the aqueous phase to achieve higher extraction yields and antioxidant activities. For hydrolysate neutralisation, we used 6 M HCl to adjust to pH 2 or concentrated formic acid to adjust to pH 3. As can be seen in Table 1, compared to pH 3, at pH 2 the TPC and DPPH^•^ scavenging activity decreased (29%, 66%, respectively), which indicated that pH influenced the protonation of the –COOH group and the electron-donating capacity of the phenolics. In addition, compared to pH 2, with the neutralised hydrolysate at pH 3 there were significantly increased levels of bound *p*-coumaric acid (25%), *trans*-ferulic acid (47%), caffeic acid derivatives (29%) and *p*-hydroxybenzoic acid (60%) (Table 1, dataset #14, #15). The increase in the concentration of some of the phenolics in the milder acidic medium might have been due to their higher stability at pH 3 than pH 2 [23]. Although the samples here were not subjected to acid hydrolysis, the alkali hydrolysates were neutralised with an appropriate amount of HCl or formic acid and stored overnight at 4 °C (until LC-MS analysis). Due to prolonged exposure, the most acid-labile phenolics might have degraded to some extent.

Our suggestion here is not consistent with Arranz and Saura Calixto [6], who reported that hydroxybenzoic, caffeic, cinammic, ferulic and protocatechuic acids were the main constituents of the sulphuric acid hydrolysate of wheat (methanol/H_2_SO_4_; 90:10 (*v/v*) at 85 °C for 20 h). In addition, they showed significantly higher levels of total phenolic acids for strong acidic hydrolysis than for alkali hydrolysis.

In the present study, for the hydrolysates neutralised with HCl and formic acid (respectively) the losses for *p*-coumaric (34%, 28%), *trans*-ferulic (32%, 21%) and *p*-hydroxybenzoic acids (28%, 18%) were higher than for the caffeic acid derivatives (42%, 45%). It is known that caffeic acid can undergo significant oxidative degradation during alkaline hydrolysis [36]. The recoveries for TPC with the hydrolysate at pH 2 were 53%, and at pH 3 they were 62%. The lower losses for individual compounds in comparison with TPC can be explained by the higher content of non-phenolic reductants and chelating agents in the relevant purified extracts. Although Folin–Ciocalteu reagent provides very useful assay, it might be non-specific and therefore not reliable. This would support Verma et al. [31], who reported that alkali hydrolysis liberated nearly twice the amounts of phenolics compared to acid hydrolysis, as determined by Folin–Ciocalteu assays. However, according to their HPLC-UV, the difference between the two protocols in terms of identifiable phenolic acids was only 21%. Those deviations were confirmed also by Irakli et al. [11], who reported that the bound phenolics content (using the Folin–Ciocalteu method; and with different cereals) were three-fold those measured using HPLC with diode-array detection.

From these results, we can conclude that with the hydrolysate adjusted to pH 3, not only were there more total and individual phenolics obtained, but also that the extract showed better scavenging activities for free radicals and better recovery of phenolics. Last, but not least, the use of formic acid is more appropriate than the use of HCl, as chlorides can corrode stainless steel flow paths, which can lead to ion contamination and pitting of the LC flow path.

To validate the influence of the sample volume loaded onto the SPE tubes on the extraction recoveries, three different loading volumes were tried (3 mL, 5 mL, 8 mL; Table 1, dataset #16, #17, #18). From Table 1, it can be seen that compared to the loaded hydrolysates of 3 mL and 5 mL (respectively), that of 8 mL showed increased TPC (77%, 79%). The DPPH^•^ scavenging activity also increased with the increase in the sample volume loaded onto the SPE tubes. In addition, and again compared to 3 mL and 5 mL (respectively), the 8 mL loaded hydrolysate showed small increases in the extracted bound *p*-coumaric acid (20%, 8%), *trans*-ferulic acid (6%, 2%) and *p*-hydroxybenzoic acid (11%, 15%). However, the content of caffeic acid derivatives with 3 mL was 16% higher than that with 8 mL (Table 1, #16 vs. #18).

Formation of hydrogen bonds between sorbent and phenolics can lead to irreversible sorption and extra difficulties in tube elution [35]. In comparison to their crude forms, it is evident that the lowest losses were seen for the 8 mL loaded samples (18%). As the volume of the loaded sample increases, the proportion of irreversibly adsorbed phenolics remains the same, consequently a higher concentration of phenolics was eluted. Therefore, the 8 mL volume was chosen as the volume for loading onto the SPE for the bound phenolic extracts.

### 3.6. Liquid-Liquid Extraction vs. Solid-Phase Extraction

Liquid-liquid extraction is the most commonly used technique for extraction of phenolic acids in cereal samples [12]. The whole LLE procedure provides three fractions, as free phenolics, soluble conjugates (combined as extractable phenolics), and bound phenolics. Instead, the direct SPE procedure provides the extractable and bound phenolics fractions (Figure 1 and Figure 2).

For increasingly sensitive chromatographic analyses, good sample preparation is essential, because it protects the chromatographic columns, and allows greater sensitivity through the removal of interfering matrix components. Selective and specific sample preparation is thus a prerequisite for reasonable, economical and sensitive analyses.

In the present study, when the extracts obtained by LLE were compared to those obtained by direct SPE purification they showed 19% lower extractable (i.e., free plus conjugated) phenolics, and 32% lower bound TPC (Figure 3a).

According to Obied et al. [37], SPE provides greater recovery of pomace phenolics than LLE. They also reported that with SPE, higher recoveries were obtained by elution with methanol than by elution with diethyl ether or ethyl acetate.

A different trend was observed in the present study for antioxidant activity, as direct SPE purification provided extracts with higher ABTS^•+^ (extractable, 29%; bound, 29%) and DPPH^•^ scavenging (bound, 3%) activities, but at the same time, the extractable phenolics were less efficient than LLE against DPPH^•^ free radicals (9%) (Figure 3b,c).

Deviations across these different tests are in agreement with Abramovič et al. [38], who reported that the number of exchanged electrons varies greatly with solvent and type of assay, whereby the majority of the compounds exchange more electrons in the Folin–Ciocalteu assays than in the ABTS and DPPH assays. According to their data, in reactions with chromogenic radicals, the numbers of electrons exchanged are higher in buffer at pH 7.4 than in MeOH (by the DPPH assay) and in water (by the ABTS assay).

**Figure 4:**
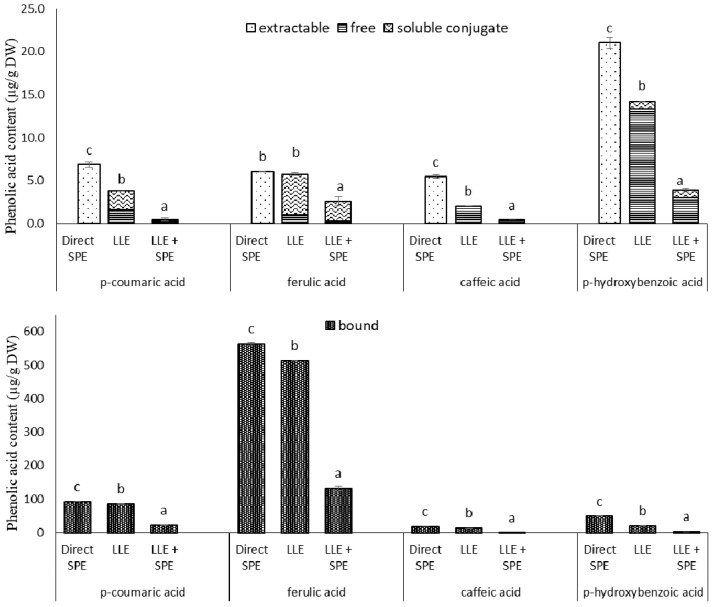
Individual phenolic acid contents from the extractions of the germinated spelt (*Triticum spelta*) seeds, as the extractable and bound phenolics from direct solid-phase extraction (SPE), and for the free, soluble conjugate and bound phenolics from the liquid-liquid extraction without (LLE) and with SPE (LLE + SPE) purification. Data are means ± SD. Different letters within the same phenolic acid as extractable and (free + soluble conjugate) or bound indicate significant differences (*p* < 0.05; Duncan’s multiple range tests).

In the present study, when compared to direct SPE, LLE with ethyl acetate showed lower levels of extractable (i.e., free plus soluble conjugates) *p*-coumaric acid (81%), caffeic acid derivatives (169%) and *p*-hydroxybenzoic acid (48%), but there were no significant differences in content of *trans*-ferulic acid (Figure 4). LLE compared to direct SPE also showed lower contents of bound *p*-coumaric acid (9%), *trans*-ferulic acid (10%), caffeic acid derivatives (38%) and *p*-hydroxybenzoic acid (144%) (Figure 4). Thus it needs to be taken into consideration that LLE is limited to partition equilibriums in the liquid phase, and requires an additional evaporation and reconstitution step. On the other hand, when using SPE, different interactions can be involved simultaneously. According to the supplier (Phenomenex) specifications, phenolics adsorption onto the SPE Strata X-RP tubes relies on three mechanisms of retention: π−π bonding, hydrogen bonding and hydrophobic interactions.

Terpinc et al. [39] showed LC chromatograms of sugars and organic acids in camelina cake extracts prior to and after passage through an SPE Strata X tube. There was also some loss of phenolics during the purification process, but their average recoveries were about 90% when a standard phenolic compound was applied to the SPE tube.

Therefore, to determine whether satisfactory purities of the extracts had already been achieved by LLE, the extracts obtained in the present study were subjected to a further purification step, by SPE (referred here to as LLE + SPE). It can be seen from Figure 4 that LLE + SPE yielded the lowest contents of the extractable and bound total and individual phenolics, as compared to the direct SPE and LLE alone procedures. Similarly, LLE + SPE yielded extracts with the lowest DPPH^•^ and ABTS^•+^ scavenging activities (Figure 3b,c). Chromatograms of LLE extracts prior to and after passage through SPE are presented in Supplementary Figures S2 and S3. Here, it can be seen that with SPE as the final purification stage following LLE (i.e., for LLE + SPE), better peak shapes can be obtained, with better separation of the different analytes from each other. Thus, LLE without this added SPE results in broader peaks and peaks with shoulders, which causes problems in the precise and accurate integration of these data.

Therefore, compared to LLE and LLE + SPE, the protocol here with direct SPE purification is the most reproducible method, and provides higher recoveries for the extractable and bound phenolics.

## 4. Conclusions

In the present study, the optimal extraction and purification techniques were determined and the efficiency of two isolation and purification methods (SPE, LLE) were compared. The optimal pretreatment conditions prior to purification of the extractable phenolics directly through SPE were the use of 99.9% methanol as the solvent, a sample-to-methanol ratio of 1:9 (*w/v*), and reconstruction in water at pH 7. The optimal extraction conditions for the bound phenolics from these germinated spelt seeds were hydrolysis with 20 mL 2 M NaOH, neutralisation with formic acid to pH 3, and purification by SPE without preliminary reconstruction. SPE was a better extraction method than LLE, as it provided greater yields of total and individual phenolics and greater antioxidant activities for both the extractable and bound fractions. Except for the removal of the matrix components, the isolation and concentration of these analytes by direct SPE was achieved in only one step. Direct SPE has emerged as an alternative to the more traditional sample preparation with LLE, particularly for the analysis of the bound phenolics. This is due to its simplicity, ease of automation, time savings, reduced use of highly toxic solvents, higher analyte recoveries, higher analyte concentrations, highly purified extracts, medium exchange when needed for subsequent analyses, and energy saving. Optimisation of sample pretreatment prior to direct SPE and optimisation of loading volumes for these SPE tubes also provides a more selective and simplified approach and reductions for the risk of errors. This purification method is recommended for use in various laboratories performing routine analyses of phenolic acids in cereal seeds. Our protocol can serve as a basis for any extraction of phenolics from different cereals, depending on the cereal matrix the protocol can be adapted. The results of the present study will contribute to the determination of bound phenolics, which are often overlooked. Future research may consider the application of different types of SPE columns.

## Figures and Tables

**Table 1:** Effects on total phenolics content (TPC), DPPH^•^ scavenging activity (DPPH) and individual phenolic acid content for the different protocols for the extractable and bound phenolics of the germinated spelt seeds.

Fraction	No.	Extraction	Extractable Fraction		Bound Fraction		In Vitro Assays (mg TE/g DW)			Phenolic Acid Content (µg/g DW)			
		Sample–to–Solvent Ratio (*w/v*)	Reconstitution	SPE (mL)	Hydrolysis: 2 M NaOH (mL)	Neutralisation	SPE (mL)	TPC	DPPH	*p*-Coumaric Acid	*trans*-Ferulic Acid	Caffeic Acid Derivatives	*p*-Hydroxy-Benzoic Acid
Crude	#1	MeOH 1:9	-	-	-	-	-	9.19	0.94	5.79	9.12	9.63	55.12
Extractable	#2	MeOH 1:9	5 mL H_2_O (pH 7)	3	na	na	na	4.10 ^b^	0.52 ^b^	3.37 ^b^	6.19 ^b^	4.68 ^a^	18.59 ^b^
	#3	H_2_O 1:9	5 mL H_2_O (pH 7)	3	na	na	na	3.95 ^a^	0.45 ^a^	3.06 ^a^	5.43 ^a^	5.72 ^b^	14.12 ^a^
	#4	MeOH 1:6	5 mL H_2_O (pH 7)	3	na	na	na	3.01 ^a^	0.46 ^a^	3.29 ^a^	6.06 ^a^	4.70 ^a^	16.78 ^a^
	#5	MeOH 1:9	30 mL 10% MeOH	30	na	na	na	3.51 ^a^	0.56 ^c^	1.97 ^a^	4.48 ^a^	4.01 ^a^	12.51 ^a^
	#6		5 mL H_2_O (pH 7)	3	na	na	na	4.10 ^b^	0.52 ^a^	3.37 ^b^	6.19 ^b^	4.68 ^b^	18.59 ^b^
	#7		5 mL acidified H_2_O (pH 3, HCOOH)	3	na	na	na	4.39 ^c^	0.53 ^b^	3.42 ^b^	5.92 ^b^	4.72 ^b^	23.62 ^c^
	#8		5 mL acidified H_2_O (pH 2, HCl)	3	na	na	na	4.47 ^c^	0.55 ^c^	3.52 ^b^	6.04 ^b^	4.79 ^b^	23.93 ^c^
	#9		5 mL H_2_O (pH 7)	3	na	na	na	4.10 ^a^	0.52 ^a^	3.37 ^a^	6.19 ^a^	4.68 ^a^	18.59 ^a^
	#10		5 mL H_2_O (pH 7)	5	na	na	na	4.28 ^b^	0.54 ^b^	3.64 ^b^	6.97 ^b^	5.10 ^b^	20.59 ^b^
Bound	#11	MeOH 1:9	na	na	10	HCOOH (pH 3)	3	6.18 ^a^	0.64 ^a^	55.19 ^a^	314.99 ^a^	7.06 ^a^	27.40 ^a^
	#12		na	na	15	HCOOH (pH 3)	3	7.57 ^b^	0.81 ^c^	64.94 ^b^	396.05 ^b^	13.04 ^b^	36.41 ^b^
	#13		na	na	20	HCOOH (pH 3)	3	7.58 ^b^	0.74 ^b^	77.33 ^c^	534.37 ^c^	21.82 ^c^	44.16 ^c^
	#14		na	na	20	HCl (pH 2)	3	7.88 ^a^	0.73 ^a^	61.96 ^a^	363.36 ^a^	16.86 ^a^	27.68 ^a^
	#15		na	na	20	HCOOH (pH 3)	3	10.20 ^b^	1.21 ^b^	77.33 ^b^	534.37 ^b^	21.82 ^b^	44.16 ^b^
	#16		na	na	20	HCOOH (pH 3)	3	7.58 ^a^	0.74 ^a^	77.33 ^a^	534.37 ^a^	21.82 ^b^	44.16 ^a^
	#17		na	na	20	HCOOH (pH 3)	5	7.49 ^a^	0.80 ^b^	86.38 ^b^	552.13 ^b^	18.00 ^a^	42.51 ^a^
	#18		na	na	20	HCOOH (pH 3)	8	13.38 ^b^	1.17 ^c^	92.92 ^c^	563.85 ^c^	18.86 ^a^	48.96 ^b^

Data are means ± SD (*n* = 3). Grouping of datasets indicate data included in the statistical analysis across each dataset. No., Numbered methodology for cross-reference with Figure 1; Figure 2; na, not applicable. SPE, loading sample on SPE tube. Different small letters within a column (TPC, DPPH, phenolic acid) indicate significant differences within the individual datasets for comparisons within the extractable and within the bound contents (*p* < 0.05; Duncan’s multiple range tests). Note: For clarity for the statistical analysis, the following samples are repeated: #2, #6, #9; #13, #15, #16.

## Data Availability

All data are contained in this article and supplementary materials.

## Supplementary Materials

The following are available online at https://www.mdpi.com/article/10.3390/antiox10071085/s1, Supplementary Table S1: Characteristics of phenolic acids from germinated spelt seeds by liquid chromatography–mass spectrometry in negative ion mode; Supplementary Figure S1: Representative HPLC chromatograms of the extractable phenolics of germinated spelt seeds in the crude extract without purification (Table 1, #1) and with purification through solid-phase extraction (Table 1, #2) (detected at 310 nm). Below: Peak times for purified sample corresponding to the defined phenolic acids determined in the present study; Supplementary Figure S2: Representative HPLC chromatograms of free phenolics of germinated spelt seeds after liquid-liquid extraction without (LLE) and with solid phase extraction (LLE + SPE) (detected at 310 nm); Supplementary Figure S3: Representative HPLC chromatograms of bound phenolics of germinated spelt seeds after liquid-liquid extraction without (LLE) and with solid phase extraction (LLE + SPE) (detected at 310 nm).
