# Supplementary material for: Development and Optimisation of Solid-Phase Extraction of Extractable and Bound Phenolic Acids in Spelt (Triticum spelta L.) Seeds
Source: Antioxidants (Basel). 2021 Jul 5;10(7):1085. doi: 10.3390/antiox10071085 (PMC8301066; doi:10.3390/antiox10071085)
Supplement: Supplementary file 1 [file antioxidants-10-01085-s001.zip › antioxidants-1265705-supplementary.pdf]

Article

Development and Optimisation of Solid-Phase Extraction of Extractable and Bound Phenolic Acids in Spelt (*Triticum spelta* L.) Seeds

Marjeta Mencin [1], Maja Mikulic-Petkovsek [2], Robert Veberič [2] and Petra Terpinc [1,*]

Table S1. Characteristics of phenolic acids from germinated spelt seeds by liquid chromatography–mass spectrometry in negative ion mode.

Phenolic acids	[M-H]- (*m/z*)	MS² (*m/z*)	Expressed as (equivalents)	Standard curve	R²	LOD (µg/g)	LOQ (µg/g)	Recovery (%)
p-Coumaric acid	163	119	*p*-Coumaric acid	y = 2327.3x	0.9911	0.176	0.534	93.49
trans-Ferulic acid	193	134, 149, 179	Ferulic acid	y = 1747.9x	0.9996	0.094	0.285	94.00
Caffeic acid	179	135	Caffeic acid	y = 1783.7x	1.0000	0.524	1.586	95.54
p-Hydroxybenzoic acid	137	93	*p*-Hydroxybenzoic acid	y = 607.04x	0.9994	0.269	0.814	89.51

LOD: limit of detection; LOQ: limit of quantitation.

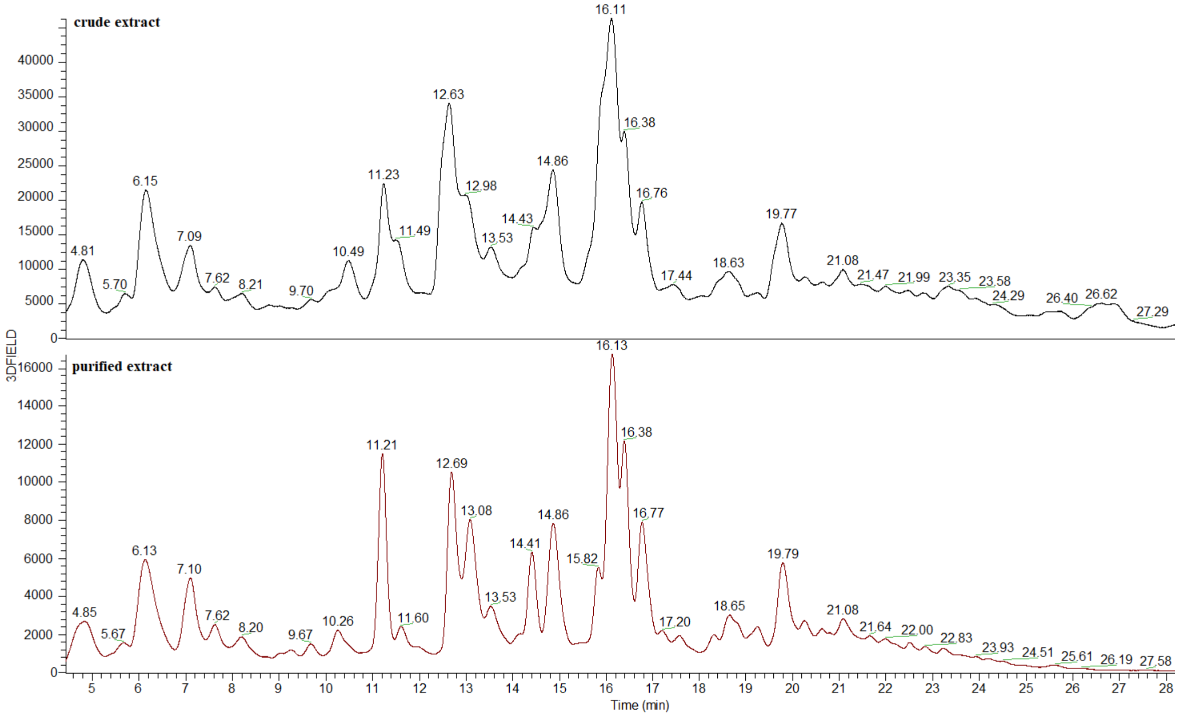

Figure S1. Representative HPLC chromatograms of the extractable phenolics of germinated spelt seeds in the crude extract without purification (Table 1, #1) and with purification through solid-phase extraction (Table 1, #2) (detected at 310 nm). Below: Peak times for purified sample corresponding to the defined phenolic acids determined in the present study.

Phenolic acid	Corresponding peak times (min)
p-Coumaric acid	18.65
trans-Ferulic acid	19.79
Caffeic acid derivatives	14.41
p-Hydroxybenzoic acid	12.69

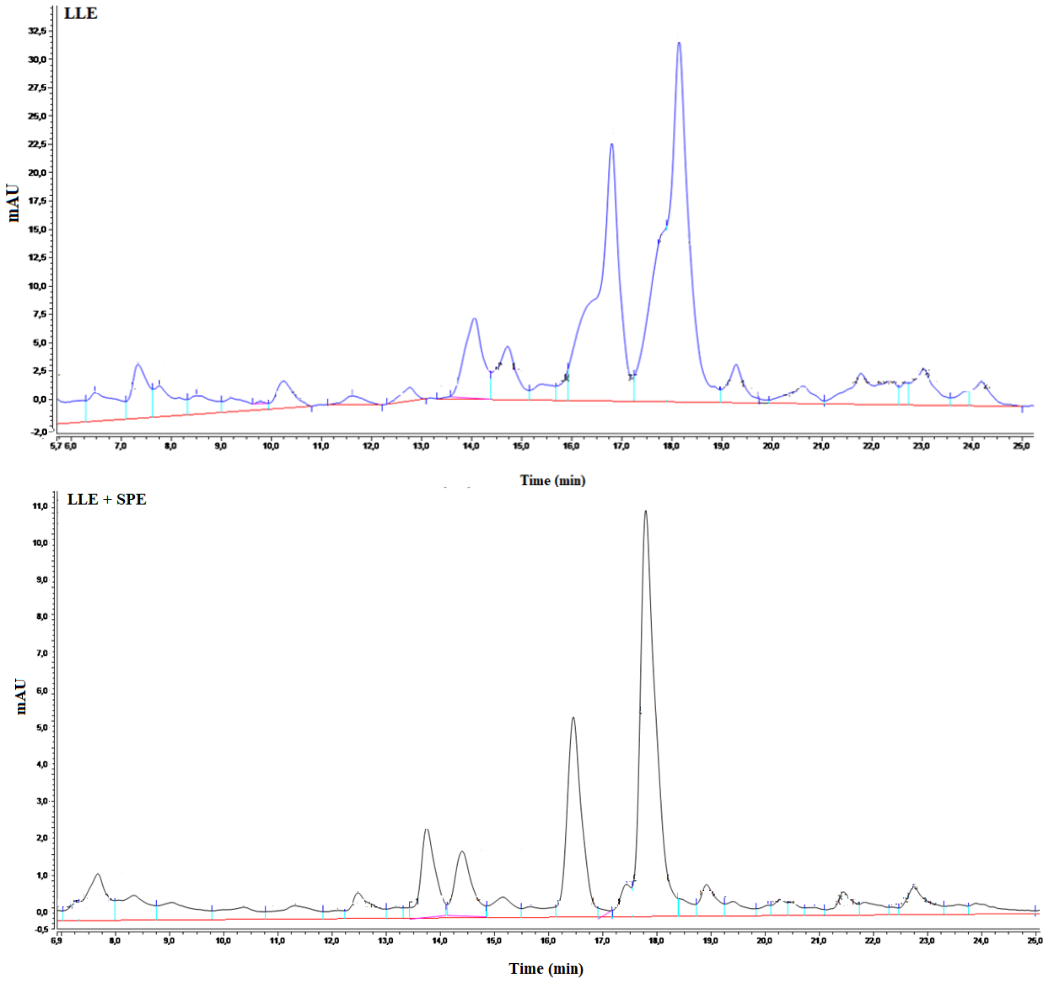

Figure S2. Representative HPLC chromatograms of free phenolics of germinated spelt seeds after liquid–liquid extraction without (LLE) and with solid phase extraction (LLE+SPE) (detected at 310 nm).

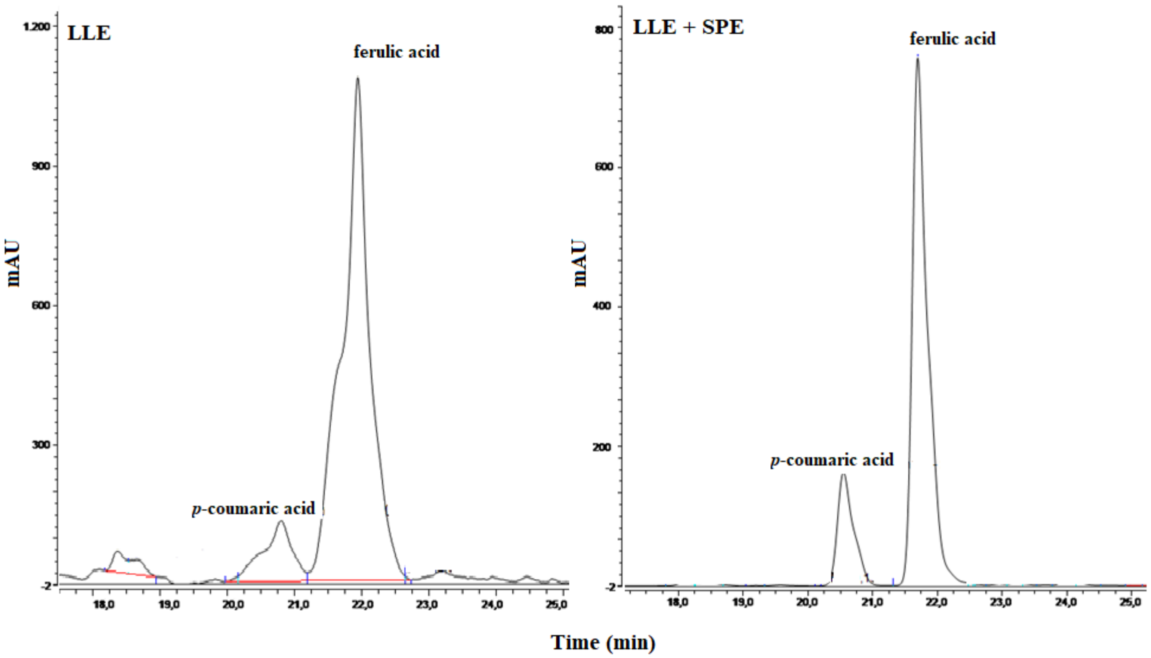

Figure S3. Representative HPLC chromatograms of bound phenolics of germinated spelt seeds after liquid–liquid extraction without (LLE) and with solid phase extraction (LLE+SPE) (detected at 310 nm).
